# Advances in the Applications of Bioinformatics and Chemoinformatics

**DOI:** 10.3390/ph16071050

**Published:** 2023-07-24

**Authors:** Mohamed A. Raslan, Sara A. Raslan, Eslam M. Shehata, Amr S. Mahmoud, Nagwa A. Sabri

**Affiliations:** 1 Drug Research Centre, Cairo P.O. Box 11799, Egypt or mohamed.raslan@pharma.asu.edu.eg (M.A.R.); info@drcbioeqs.com (S.A.R.); eslam.mansour@drcbioeqs.com (E.M.S.); 2 Department of Obstetrics and Gynecology, Faculty of Medicine, Ain Shams University, Cairo P.O. Box 11566, Egypt; amrsaadobygyn@med.asu.edu.eg; 3 Department of Clinical Pharmacy, Faculty of Pharmacy, Ain Shams University, Cairo P.O. Box 11566, Egypt

**Keywords:** chemoinformatics, bioinformatics, applications, formulation, advances

## Abstract

Chemoinformatics involves integrating the principles of physical chemistry with computer-based and information science methodologies, commonly referred to as “in silico techniques”, in order to address a wide range of descriptive and prescriptive chemistry issues, including applications to biology, drug discovery, and related molecular areas. On the other hand, the incorporation of machine learning has been considered of high importance in the field of drug design, enabling the extraction of chemical data from enormous compound databases to develop drugs endowed with significant biological features. The present review discusses the field of cheminformatics and proposes the use of virtual chemical libraries in virtual screening methods to increase the probability of discovering novel hit chemicals. The virtual libraries address the need to increase the quality of the compounds as well as discover promising ones. On the other hand, various applications of bioinformatics in disease classification, diagnosis, and identification of multidrug-resistant organisms were discussed. The use of ensemble models and brute-force feature selection methodology has resulted in high accuracy rates for heart disease and COVID-19 diagnosis, along with the role of special formulations for targeting meningitis and Alzheimer’s disease. Additionally, the correlation between genomic variations and disease states such as obesity and chronic progressive external ophthalmoplegia, the investigation of the antibacterial activity of pyrazole and benzimidazole-based compounds against resistant microorganisms, and its applications in chemoinformatics for the prediction of drug properties and toxicity—all the previously mentioned—were presented in the current review.

## 1. Introduction

Chemoinformatics, a new area of information technology, is primarily concerned with collecting, retaining, examining, and reorganizing chemical information. Small molecule formulae, structures, characteristics, spectra, and activities (biological or industrial) are typical examples of chemical data of interest. It began as an aiding tool in the process of drug discovery and development; however, presently, its significance has grown multifold, making it an essential component in numerous domains of chemistry, biochemistry, and biology [1].

The identification of hits is the first and most important stage in small-molecule drug discovery [2]. The employment of virtual chemical libraries in diverse virtual screening methods has become a promising approach to discovering novel hit chemicals. In this regard, several scholars are developing innovative de novo chemical and on-demand libraries using various in silico methodologies [3].

**Figure 1:**
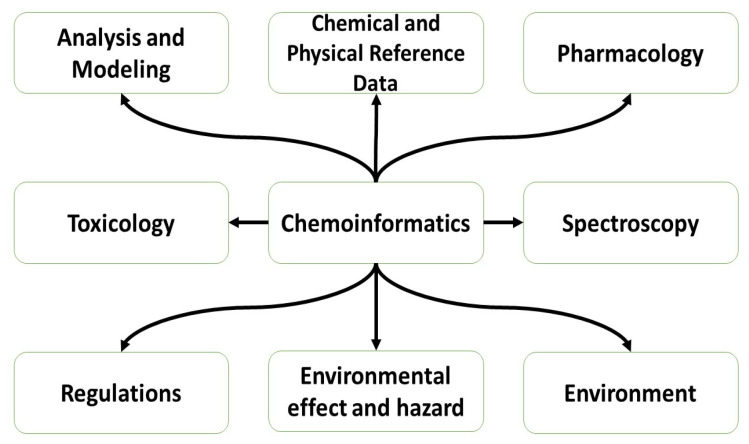
Chart presenting machine learning in chemoinformatics and drug discovery [4].

The chart (Figure 1) showed that chemoinformatics analysis involves a computational workflow utilizing machine learning. The process includes the following steps: The initial step involves extraction involving compound characterization by its substructure fragments or other chemical descriptors. Representation of the chemical features of the compound by chemical fingerprints, which are then used to compare the similarities between different compounds based on shared chemical features. Moreover, these chemical fingerprints can be utilized in various machine learning models, including instance- and/or model-based learning, to predict other chemical and physiochemical properties in QSAR/QSPR analysis. Such models can be trained using statistical models and then used to make inferences from the training data by comparison [4].

In general, virtual libraries address the requirement for increasing compound quality in order to discover promising compounds. In this context, the virtual libraries’ structural complexity, size, and variety are important factors in boosting the likelihood of favorable outcomes in drug discovery and development. Moreover, the establishment of virtual libraries is of immense advantage as the identified chemicals possess a certain degree of novelty and are synthetically viable [5]. There are several methods for creating a virtual chemical compound, including employing a known reaction schema and readily available chemicals, de novo-based design, morphing/transformation, or painting a molecular graph [6].

It is worth mentioning that both metabolism and conveyance are important factors in determining a molecule’s bioavailability and biological activity. Keeping organized and reliable experimental data in a suitable repository as a relational database promotes straightforward computer processing and hence allows computational analyses to effectively infer high-quality information/knowledge. Metrabase is an exemplary database that combines both cheminformatics and bioinformatics resources, including thoroughly examined data on the transportation and metabolism of chemical substances in humans. Its major components consist of around 11,500 instances of interaction involving almost 3500 small molecule substrates and transport protein modulators, as well as CYP450 metabolic enzymes [7].

From the aforementioned, it is clear that bioinformatics and chemoinformatics are becoming essential with the continuous growth of both biological and chemical data, as these fields have the potential to revolutionize the life sciences and make a significant impact on human health. Understanding and developing new methods and tools that can be used to identify new drug targets, develop new diagnostic tests, and track the spread of diseases, as well as helping scientists better understand and manage biological and chemical data.

**Figure 2:**
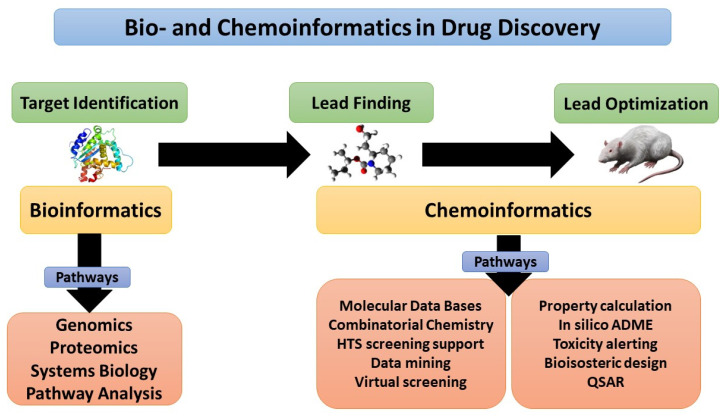
Drug discovery’ steps using Bio- and chemoinformatics tools [8].

Figure 2 showed that bioinformatics and chemoinformatics tools are both complementary to each other in the drug discovery journey, where target identification represents the initial step in this journey, which can be done by various tools such as genomics and proteomics. The lead finding and optimization can be performed by several tools, such as data mining, QSAR, and insilico-ADME, where the resulting product is an active medicinal molecule that provides therapeutic response with low or minimal adverse effects [8].

## 2. Materials and Methods

The following sources were considered in the current review: retrospective and prospective comparative cohort studies, randomized controlled trials, case studies, reviews, controlled non-randomized clinical trials, and systematic reviews.

**Figure 3:**
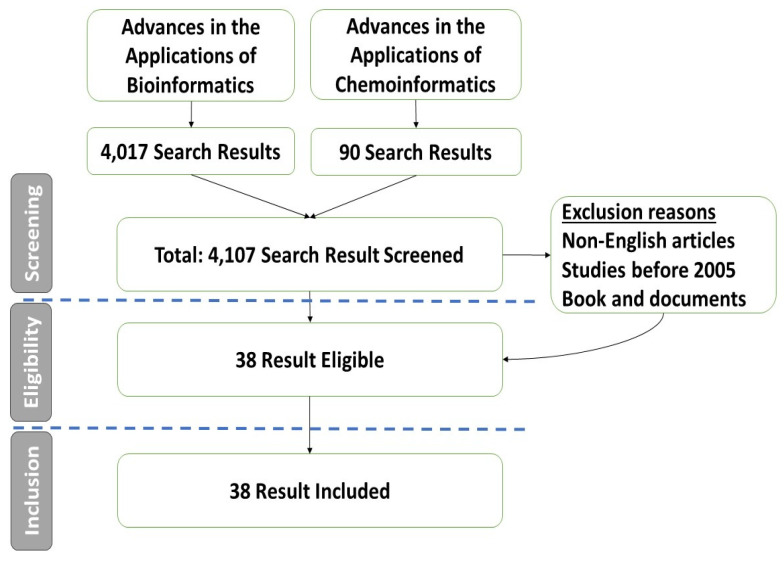
Articles’ selection Flowchart.

The search strategy employed medical subject headings (MeSH) to ensure efficient retrieval of relevant scholarly articles. The MeSH terminology of chemoinformatics, bioinformatics, antimicrobial medications, and Egypt was used to search PubMed and MEDLINE databases. Only studies in the English language were included. All relevant publications up to 2023 were included (Figure 3). No specific constraints were imposed on the search in terms of the design of the study. Following the removal of duplicate studies from our study pool, each of the included studies underwent a rigorous screening process against both the inclusion and exclusion criteria. The primary focus of the inclusion criteria was scrutinizing published literature, which evaluated the recent applications of bio- and chemoinformatics.

## 3. Drug Discovery and Design

### 3.1. Chemoinformatics and New Tetracycline Analogue

Antimicrobial resistance to existing antibiotics indicates a critical global crossroads [9]. Unfortunately, widespread antibiotic use has resulted in the emergence of multi-drug-resistant pathogenic organisms and a reduction in the efficacy of many of our most potent antibiotics [10]. In addition, various harmful consequences of antibiotics, most notably the rising prevalence of Clostridium difficile-associated inflammatory bowel disease were investigated [11].

Tetracycline, a bacteriostatic agent, has the ability to inhibit the growth of a diverse array of microorganisms, encompassing Gram-negative and Gram-positive bacteria, mycoplasmas, chlamydiae, and rickettsiae [12]. The mechanism of bacterial resistance to tetracycline antibiotics includes mutations within the ribosome binding site or the acquisition of mobile genetic elements containing tetracycline-specific resistance genes [13]. The process of protein synthesis can be hindered by the binding of tetracycline to the 30S ribosomal subunit, which ultimately prevents aminoacyl transfer RNA (tRNA) from accessing the acceptor site on the ribosome [14].

The in vitro antibacterial activity of a new tetracycline analogue generated semi-synthetically from Streptomyces species was investigated to determine the minimum inhibitory concentrations (MICs) for the growth of several harmful bacteria. The chemo-informatics approach was used to create tetracycline analogue B (iodocycline), which was more active as a bacteriostatic antibacterial agent than tetracycline and thus had less bacterial resistance. In comparison to the chloramphenicol prototype antibiotic, tetracycline analogue B has MICs of less than 10 micrograms/mL for bacterial growth, indicating its potent antibacterial action [15].

### 3.2. Bio- and Chemoinformatics in Identification of Novel Pyrazole and Benzimidazole Based Derivatives as Penicillin-Binding Protein 2a Inhibitors

Methicillin-resistant Staph aureus’s (MRSA’s) extensive resistance to the lactam class is associated with the characteristics of its primary resistance mechanism, the “acquired” penicillin-binding protein 2a (PBP2a). The PBP2a’s innate reduced sensitivity towards β-lactam inactivation is attributed to its affinity for a closed active-site conformation, regulated by allostery [16]. PBP2a may cross-link the cell wall even when β-lactam antibiotics are present, whereas the other four native PBPs are restrained [17].

A research project involved the development, synthesis, and examination of ten compounds based on pyrazole and benzimidazole to investigate their antibacterial activity against two strains of Staphylococcus aureus, namely, MSSA ATTC6538 and MRSA USA300. The findings revealed that three of the investigated compounds showed modest bactericidal efficacy against MRSA, VRSA, and MSSA. Furthermore, the compounds were docked into the allosteric region of PBP2a and exhibited binding patterns similar to those of the lead quinazolinone PBP2a inhibitors, suggesting a comparable mechanism of action [18].

### 3.3. Chemoinformatics Application in Phytochemistry

Natural products are thought to be a promising source of antifibrotic medicines; however, finding and isolating bioactive molecules remains difficult. The good news is that various computational approaches have emerged on this subject to save time and effort [19].

Eucalyptus globulus Labill., a perennial tree belonging to the family Myrtaceae, is widely cultivated across the globe. Eucalyptus species are commonly planted as line plantings in Egypt for multiple purposes, including shade provision, building timbers, poles, and fuelwood. One of the most significant byproducts in the Eucalyptus industry is its bark. Eucalyptus bark is thought to be an excellent source of phenolic chemicals with a variety of biological activities [20,21].

Polyphenols have a variety of uses in the cosmetics, food, and pharmaceutical sectors. This group of chemicals has been shown to have antioxidant, antimicrobial, antidiabetic, anti-inflammatory, antihyperlipidemic, hepatoprotective, nephroprotective, cardioprotective, and anticancer properties [22].

In the course of a research project, the chemical and biological characteristics of Eucalyptus globulus bark were determined by the use of Sirius software, and 37 compounds were tentatively identified; 15 of them were newly discovered from this particular species. In addition, the bio-transformer tool was proficiently applied to conduct an in silico virtual assessment of the human metabolism of these substances, resulting in the generation of a total of 1960 unique products through diverse metabolic pathways. In addition, an electronic database of the discovered chemicals and metabolites was generated and subjected to in silico docking against eight protein targets that are known to be involved in the liver fibrosis process. The findings suggest that the extract may have a hepatoprotective impact via many pathways and that the metabolites have been found to have stronger affinities towards the relevant enzymes when compared to their parent chemicals. The extract demonstrated significant cytotoxicity against the hepatic cancer cell lines HEPG2 and HUH-7, and its cellular uptake was enhanced through nano-formulation, as demonstrated by the ex vivo everted gut sac technique [19].

## 4. Clinical Applications

### 4.1. Bioinformatics and Heart Disease Classification

For decades, heart disease has been regarded as the primary factor contributing to global death rates. In 2016, the World Health Organization reported that a sizable number of 17.9 million individuals had passed away due to cardiovascular disease [23]. Thus, data mining technologies have been investigated in recent decades to enhance heart disease prediction processes in the medical field [24].

The practice of discovering hidden patterns, information, and anomalies in massive data sets is known as data mining, which is regarded as the central component in the knowledge discovery in databases (KDD) process, which includes a number of phases such as data preparation, selection, transformation, and mining, which entails diverse activities such as prediction, clustering, and classification [25,26].

A quantitative study using the ensemble model in conjunction with brute force as a technique for selecting features to classify heart diseases resulted in a remarkable accuracy rate of 97.8%. The suggested stacking model has been demonstrated to be efficient and outperforms existing techniques in the categorization of cardiac disorders [27].

### 4.2. Bioinformatics and Diagnosis of Coronavirus Disease 2019

The outbreak of COVID-19 has posed a significant threat to the lives and well-being of many people, causing confusion in the global population’s public life. The escalating number of COVID-19 cases showed that all countries were faced with the daunting challenge of depleting resources for virus detection. The unprecedented spread of the virus has placed an immense strain on the limited resources available for the detection of this highly infectious disease. In order to effectively combat the spread of COVID-19, it is imperative to implement a COVID-19 detection system that is readily available, cost-effective, and capable of automation [28].

Due to the widespread presence of radiology imaging equipment in medical facilities, radiography-based detection techniques have emerged as a viable detection method to resolve the shortage of virus testing kits. With the advent of machines and deep learning, artificial intelligence has become highly advanced and thus fundamental in the field. As such, leveraging these technologies in radiography-based testing methods can offer an efficient and effective means of detecting COVID-19 [29].

Deep learning techniques for the purpose of automated COVID-19 identification and categorization are being widely investigated [30]. As a result, deep learning has emerged as a critical component of automated clinical decision-making [31].

A study for the diagnosis of COVID-19 using Chest CT and X-ray images provided multi-classifiers rather than a single classifier layered in an ensemble stacking manner. When applied to datasets consisting of X-ray pictures and CT scans, the findings showed a quantitative evaluation of the suggested ensemble stacking technique, with percentages approaching 99% [32].

**Figure 4:**
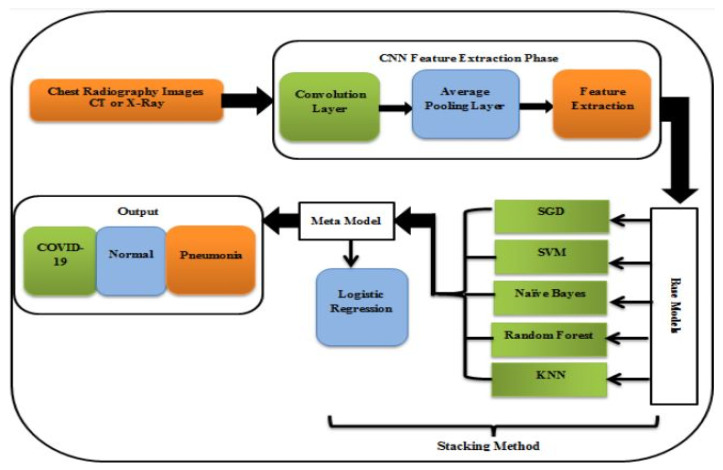
Model for COVID-19 detection [32].

Figure 4 represents the utilization of a COVID-19 detection stacking methodology that comprises two models as follows: The first (base) model is comprised of five classifiers: SGD, SVM, naive bayes, random forest, and KNN. The reason for selecting five classifiers is to ensure that there is always a majority identification, as opposed to using an even number of classifiers, which could result in an equal division of outcomes between two categories. The second model, referred to as the meta model, is logistic regression. This two-tiered approach to detecting COVID-19 is expected to yield more accurate results compared to using a single model alone [32].

### 4.3. Bioinformatics and Genomic Correlation with Clinical Information and Disease State

A PCR-based analysis has established a correlation between obesity and specific polymorphisms, including UCP2 G 866 A, LEPR Gln223Arg, and INSR exon 17, with the added observation that certain variations of risk are influenced by gender [33].

*Additionally, a research study using an Illumina short-read sequencer-based investigation of the entire genomes of nine Egyptian women showed that 12 SNPs were shared by the majority of the participants related to obesity and were concordant with their clinical diagnosis using 30x sequencing coverage. Also, the presence of the mtDNA mutation A4282G in all samples was reported.*

[34]

### 4.4. Bioinformatics and Multiple Drug Resistant Escherichia coli (E. coli) Isolation from Pediatric Cancer Patients

*Escherichia coli* is the primary etiological organism responsible for the incidence of bloodstream and urinary system infections globally. A steady growth in *E. coli* antibiotic resistance affects medical institutions worldwide by creating difficult-to-treat infections in patients [35]. Multiple drug resistance (MDR) genetic patterns are widely found in mobile elements like transposons, integrons, and plasmids that are passed on from foodborne pathogens to human pathogens, boosting their pathogenicity [36].

The emergence of next-generation sequencing (NGS) has opened up new possibilities for efficient characterization of bacterial infections, enabling the identification of virulence-associated factors and genes that mediate resistance to antibiotics [37]. It is worth mentioning that NGS is a widely used technology for studying the evolutionary connections of MDR *E. coli* strains from various geographical locations; thus, through the analysis of genetic variations in diverse *E. coli* plasmids obtained from multiple sources, it is plausible to anticipate resistance traits from genomic sequences [38].

Quinolones and aminoglycoside resistance were observed in 21 carbapenem-resistant *E. coli* (CRE) isolates by using the Illumina next-generation sequencing platform for plasmid shot-gun sequencing and data analysis with the bioinformatics pipeline. The highest represented genes among the 32 antimicrobial resistance genes discovered were the aph(6)-Id gene, sul2, aph(3′)-Ia, sul1, dfrA12, aph(3″)-Ib, NDM-11, and TEM-220. Out of all the isolates, only two of them exhibited virulence factors that were linked with the iroA gene cluster, and it was found that this gene cluster was present in only one of those two isolates. The findings indicate that there is a potential for the transfer of resistance genes and plasmids between species beyond the scope of nosocomial infections among hospitalized patients [39].

## 5. Optimization of Drug Delivery

### 5.1. Bio- and Chemoinformatics in Nose-To-Brain Formulation Targeting Meningitis

Meningitis is a serious medical condition caused by a diverse range of pathogens that can result in death. The meninges become infected or inflamed due to various infectious agents. This condition can be caused by a diverse range of pathogens [40]. It has been observed that viruses are responsible for nearly 50% of all cases, whereas fungi, usually cryptococci, are accountable for less than ten percent of all cases [41]. Bacterial meningitis is an illness that is considered to be the most severe type of meningitis. The majority of infections in newborns are caused by Group B. Streptococcus agalactiae, Listeria monocytogenes, and *Escherichia coli*, as well as Haemophilus influenzae, have been associated with bacterial meningitis, with the highest incidence in children under five years of age. Despite the availability of antibiotics, acute bacterial meningitis is a major cause of morbidity and mortality. Survivors are at risk of long-term repercussions such as brain damage, hearing loss, and learning impairments [42].

Bio- and chemoinformatics methods were used for comparative analysis of antimicrobial drugs to choose an effective nasal-to-brain delivery formulation that targets meningitis, where it was found that cephalosporin antibiotics, namely, cefotaxime and ceftriaxone, were comparable concerning formulation, biopharmaceutical, and therapeutic levels. An all-atom approach was employed for molecular dynamics simulations using the GROMACS v4.6.5 software, and the results showed that ceftriaxone has a higher affinity for the biopharmaceutical and therapeutic macromolecules studied than cefotaxime [43].

Additionally, cefotaxime and ceftriaxone docked successfully on the P-gp efflux pump, S. pneumoniae PBP1a and 2b, and mucin, showing that ceftriaxone exhibited a greater level of affinity towards the P-gp efflux pump and docked more successfully on mucin, while on the gelatin and tripalmitin matrices, ceftriaxone showed decreased out-of-matrix diffusion and increased trapping compared to cefotaxime. Thus, the use of ceftriaxone gelatin nanospheres and tripalmitin solid lipid nanoparticles as a nose-to-brain formulation aimed at treating meningitis could potentially offer a more feasible and effective approach than cefotaxime [43].

### 5.2. Chemoinformatics Targeting Cancer Cell Therapy

Carcinogenesis is a complicated process involving the interplay of various elements that lead to an alteration in regular cellular functions and the eventual transformation of cells into a malignant state [44].

A comprehensive analysis of the various functions of the interacting components within the tumor microenvironment is crucial in the fight against cancer, which could lead to a better understanding of this unfavorable cell transformation and, as a result, the identification of potential molecular targets for early prognosis together with the discovery of chemotherapeutic drugs [45].

**Figure 5:**
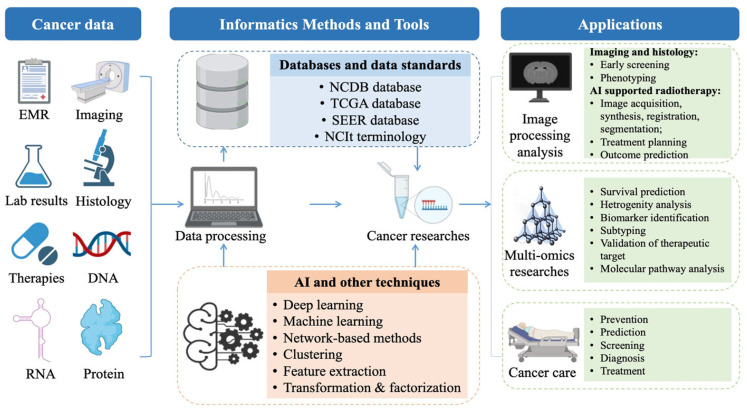
Summary of Cancer Informatics [47].

Epithelial cell transforming 2 (ECT2) is a putative oncogene that has been linked to the advancement of numerous human malignancies in recent investigations. Despite the increased interest in ECT2 in oncology-related papers, there has to be a thorough examination that consolidates and harmonizes the expression and oncogenic conduct of ECT2 across a range of human malignancies. Using numerous databases, ECT2 could potentially function as a valuable biomarker across an array of malignancies; thus, chemoinformatics was used to investigate which ECT2 inhibitors might be used as anticancer medicines r. In addition, it was found that ECT2 was overexpressed in both mRNA and protein forms in different types of tumors, causing an increased infiltration of myeloid-derived suppressor cells and a decrease in the levels of natural killer T-cells, resulting in a poor prognosis for survival [46]. Figure 5. Presents a summary of cancer informatics which showed that the incorporation of a range of informatics techniques and instruments makes it possible to scrutinize diverse cancer data and the application of artificial intelligence (AI) algorithms holds the promise of enhancing desired therapeutic outcomes [47].

(a) The incorporation of a range of informatics techniques and instruments makes it possible to scrutinize diverse cancer data and generate approaches for preventing, screening, diagnosing, and treating the disease.
(b) The application of artificial intelligence (AI) algorithms holds the promise of enhancing desired therapeutic outcomes. The benefits of AI tools in interpreting medical images have been established in various environments and for a range of diseases.
(c) This technology could be utilized to analyze data from multiple sources to identify patterns and early warning signs of cancer, thereby enabling prompt intervention and more effective treatment.

### 5.3. Bio- and Chemoinformatics in Nose-To-Brain Formulation for Treatment of Alzheimer Disease

It is worthy of mention that delivering drugs to the brain for treatment of severe CNS illnesses such as Alzheimer’s has remained a significant issue for pharmaceutical formulation and development. This is primarily attributed to the numerous defense systems against drugs’ delivery to the brain. These systems present formidable barriers that most drugs are unable to overcome, making it difficult for them to cross the blood–brain barrier and penetrate the extracellular matrix of the brain to reach the targeted brain cells [48].

As a result, while directing medications to the brain poses a significant obstacle in the treatment of many CNS illnesses, a novel route of administration looked promising in tackling this problem. This is known as ‘Nose-to-Brain’ targeting. Recent investigations have shown that if the medication is delivered intranasally, a part of it can skip the blood–brain barrier (BBB) and enter the brain directly, which occurs via the olfactory and trigeminal nerve systems [49].

A research work proposes a novel approach to evaluating two natural compounds, curcumin and its congener bisdemethoxycurcumin (BDMC), aiming to identify a potential nose-to-brain treatment for Alzheimer’s disease. It was found that curcumin outperformed BDMC. Moreover, five novel analogues were also proposed, with diethoxybisdemethoxycurcumin being chosen as the best, and thus, it was proposed that the use of bio/chemo informatics tools be used as a dependable, cost-effective alternative to time-consuming, resource-intensive laboratory work [50].

## 6. Some Advances in New Algorithms and Artificial Intelligence Worldwide

### 6.1. Chemoinformatics and Hybrid Harris Hawks Optimization with Cuckoo Search

One of the significant problems in cheminformatics is the large datasets containing a significant amount of redundant information. This redundancy can negatively impact similarity measurements with respect to drug design and discovery, which could be solved through a hybrid metaheuristic algorithm called CHHO–CS that combines the Harris–Hawks optimizer (HHO) with two operators, cuckoo search (CS), and chaotic maps to balance exploration and exploitation phases and avoid premature convergence. The experimental and statistical analyses demonstrate that the CHHO–CS method outperforms competitor algorithms such as HHO, CS, particle swarm optimization, etc. The proposed algorithm is expected to improve the efficiency and accuracy of similarity measurements for drug design and discovery [51].

### 6.2. Chemoinformatics and Bioinformatics Integration with Artificial Intelligence (AI)

The insufficiency in effectiveness resulting from issues related to the availability of the drug in the body and unfavorable reactions to the drug are acknowledged as a primary reason for the termination of clinical trials. The vast array of potential factors that may lead to the failure or adverse effects of a compound is expansive. Additionally, the assessment of a compound’s characteristics through in vitro and in vivo methods can be a significant investment in terms of both time and resources. As a result, extensive endeavors have been undertaken to devise computational models that can anticipate absorption, distribution, metabolism, excretion, and toxicity (ADME-Tox) properties [52]. These efforts are driven by the need to streamline and improve the process of drug development, especially with regard to the identification of potential risks associated with new compounds.

The application of AI models has made significant strides in enhancing the precision of early drug efficacy and safety predictions by leveraging the vast information provided by heterogeneous ADME-Tox data sets. In recent times, there has been a surge in both public and private sector initiatives seeking to create and assess prospective models that would aid decision-making processes and generate innovative approaches for predicting ADME-Tox properties. As a result, there are ongoing efforts to allow for the public use and comparison of machine learning (ML)/deep learning (DL) models to bolster confidence and acceptance of these predictions. An example of this is the Therapeutics Data Commons (TDC), which offers a platform for systematic access and evaluation of ML models across the entire range of therapeutics through an open Python library [53,54].

In the domain of machine learning (ML), various models have been developed to derive hypothetical properties from limited experimental data or to characterize in vivo properties based on in vitro assay data. However, there are potential limitations to the accuracy of such models. In this regard, Rodríguez-Pérez et al. demonstrated the effectiveness of multitask learning based on graph neural networks (MT-GNN) in achieving superior performance compared to other ML approaches that rely solely on in vitro brain penetration data [55].

There are four areas in computational biology where ML and DL can be integrated with established bioinformatic methods, namely: molecular evolution, protein structure analysis, systems biology, and disease genomics. In addition, machine learning algorithms such as support vector machines (SVM), K-nearest neighbors (KNN), convolutional neural networks (CNN), recurrent neural networks (RNN), principal component analysis (PCA), t-distributed stochastic neighbor embedding (t-SNE), and non-negative matrix factorization (NMF) are frequently used in bioinformatics research [56].

**Figure 6:**
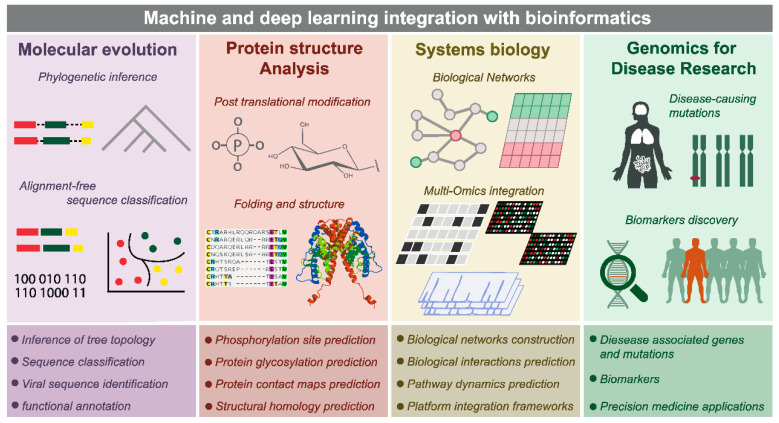
Bioinformatics Applications of Integrated Machine Learning Techniques [56].

Figure 6 shows that the utilization of integrated machine learning techniques in combination with bioinformatics has proven to be a valuable tool in various fields, namely molecular evolution, protein structure analysis, systems biology, and disease genomics. Molecular evolution includes alignment-free sequence classification and phylogenetic interference. Protein structure analysis includes post-translational modifications. Systems biology includes biological networks and multiomics integration. Disease genomics includes disease-causing mutations and biomarker discovery. The end goal of bioinformatics applications integrated with machine learning is to provide precision medicine applications for each individual case.

Integrating machine learning into molecular evolution research has enabled accurate determination of evolutionary distances between species, reconstruction of evolutionary histories and ancestries, identification of conserved genomic regions, functional annotation of genomes, and phylogenetics [56]. Methods such as autoencoders, random forests [57], convolutional neural networks (CNNs) [58], and deep reinforcement learning [59] have been used to address the challenges faced by molecular evolution research, particularly in analyzing increasingly massive sets of sequence and other omics data [60].

Machine learning techniques have been integrated with traditional proteomic methods to predict and analyze post-translational modifications, including CNN, hierarchical clustering, and K-means clustering. Ensemble Gly developed an ensemble classifier of protein glycosylation sites based on a curated glycosylated protein database and SVM. Several DL models have been incorporated with other modeling techniques and curated databases for the prediction of phosphorylation sites and protein glycosylation [56].

Moreover, system biology is used with ML to analyze complex omics datasets, integrate different data types, model complex interactions, and model biological systems. ML methods in network biology can be classified into those that infer the network architecture and those that integrate existing network architectures with biological data measurements. These techniques require sophisticated data integration methods to incorporate different data types into a model [61].

On the other hand, genomics and biomarker analysis for disease research are integrated with ML to identify disease-associated genes and mutations for diagnosis, predict disease progression and clinical outcome, and enable personalized medicine. Different applications include the identification of genes associated with complex diseases, the analysis of complex omics datasets, and the prediction of protein glycosylation and phosphorylation sites. Moreover, ML techniques have been used to address the key challenges in disease research, which include the identification of disease-associated genes and mutations for diagnosis, prediction of disease progression and clinical outcome, drug response, and personalized medicine [56].

Table 1 represents the integration of machine learning techniques with bioinformatics tools applied to address various representative issues in four key domains: molecular evolution, protein structure analysis, systems biology, and biomarker analysis for disease research. In each main area, the problem is categorized. Furthermore, the target goals, bioinformatic tools, and machine learning methods are identified.

Table 2 represents the different chemo/bioinformatics applications, including antibiotic discovery, disease diagnosis and classification, phytochemistry therapeutic discovery, cancer cell targeting, special pharmaceutical formulation, identification of multidrug-resistant organisms, genomic correlation with disease state, and artificial intelligence integration.

**Figure 7:**
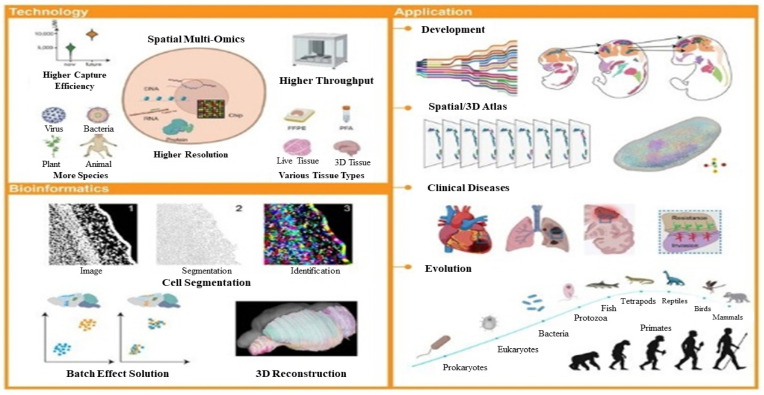
Future perspectives of bioinformatics, and spatially resolved transcriptomics related technology [89].

Figure 7 shows the future of SRT-related technology, bioinformatics, and their applications, which are of great interest and importance in the scientific community. The ongoing advancements in SRT technologies and bioinformatics algorithms have been instrumental in accelerating research in fields such as embryonic development, spatial atlases, clinical diseases, and evolution. These developments have the potential to profoundly impact both basic science and translational medicine, leading to breakthroughs in the diagnosis, prevention, and treatment of diseases as well as advancements in our understanding of fundamental biological processes [89].

## 7. Conclusions

Chemo- and bioinformatics showed different applications globally in research studies. The use of virtual chemical libraries and virtual screening methods can increase the probability of discovering novel hit chemicals. The outcomes include several benefits in drug discovery, disease diagnosis and classification, special pharmaceutical formulations for minorities and Alzheimer’s disease, and phytochemistry therapeutic discovery. Ensemble models and brute force feature selection methodology have resulted in high accuracy rates for heart disease and COVID-19 diagnosis. Other benefits of pharmaceutical research include targeted cancer cell therapy, the identification of novel molecules for antimicrobial resistance, genomic correlation with disease state, and the identification of multidrug-resistant organisms. Moreover, the use of AI in chemoinformatics can help in the prediction of drug properties and toxicity, while AI in bioinformatics can aid in the analysis of large-scale genomic and proteomic data. It is essential to extend the application of chemoinformatics in drug discovery, clinical pharmacy settings, and the formulation of targeted dosage forms for special diseases, as there is no broad use of chemoinformatics in these areas.

## Figures and Tables

**Table 1:** Implementation of machine learning techniques in bioinformatics to address representative problems and explore the effectiveness of such methods.

Reference	Problem Category	Goal	Bioinformatic Tools	ML Method	Bioinformatics Area
[62]	Biological sequence clustering	Protein family prediction	Clusters of Orthologous Groups (COGs) and G protein-coupled receptor (GPCR) dataset	CNN	Molecular evolution
[63]		Protein function prediction	BLAST and HMMER search	deep RNN	
[64]		Anti-CRISPR proteins identification	MSA and PSI-BLAST	Random forest	
[65]			K-mer based clustering (CD-HIT), BLAST	EXtreme Gradient Boosting	
[66,67]		Viral pathogenicity feature identification	MSA, phylogenetic tree construction	SVM	
[68]	Alignment free biological sequence analysis	Identification of viral genomes	BLAST, Sequence clustering, HHPRED	RNN	
[69]			BLAST	CNN	
[70]	Post translational modifications	Phosphorylation sites prediction	Local sequence similarity	KNN	protein structure analysis
[71]			K-mer based clustering (CD-HIT), BLAST	CNN	
[72]		Glycosylation sites prediction	curated glycosylated protein database (O-GLYCBASE)	ensemble SVM	
[73]	Protein structure prediction	Protein contact prediction	MSA	CNN	
[74]		Prediction of distances between pairs of residues	MSA, HHPRED, PSI-BLAST	CNN	
[75]	inference of biological networks	Gene regulatory network prediction	GeneNetWeaver, RegulonDB	SVM	systems biology
[76]		Protein-protein interaction network prediction	Domain affinity and frequency tables	SVM	
[77]			Protein descriptors	Elastic-net regression	
[78]	Analysis of biological networks	Drug target prediction	Network analysis tools	K-means	
[79]		Drug side effect prediction	Genome scale metabolic modeling	SVM	
[80]		Drug Synergism prediction	A chemical-genetic interaction matrix	Random Forest Ensemble	
[81]	Multi-omics integration	Cancer subtype prediction	Similarity based integration	Neighborhood based clustering	
[82]		Drug response prediction	Cancer hallmarks datasets, pathway data	logistic regression	
[83]	Disease-associated genes investigation	Pulmonary sarcoidosis genes identification	Differential expression analysis	Hierarchical clustering	biomarker analysis for disease research
[84]		Identification of miRNA-disease association	Disease semantic information and miRNA functional information	NMF	
[85]		Disease-phenotype visualization	OMIM database and human disease networks	t-SNE	
[86]	Biomarker discovery	Cancer diagnosis	Reference gene selection	SVM	
[87]		Biomarker signature identification	Network-based gene selection	SVM	
[88]		Cancer outcome prediction	Evolutionary conservation estimation	Random forest	

**Table 2:** Summarized Information about Chemo/Bioinformatics Applications.

Reference	Informatics Used	Application	Outcome
[15]	Chemoinformatics	Antibiotic discovery	Tetracycline analogue B (iodocycline). More active than tetracycline and less bacterial-resistant.
[27]	Bioinformatics	Disease Classification	The implementation of the ensemble model, in conjunction with brute force as a feature selection methodology, results in an exceptional accuracy rate of 97.8% for the categorization of heart disease.
[32]	Bioinformatics	Disease Diagnosis	Based on data from X-ray pictures and a CT scan, the findings showed a quantitative evaluation of COVID-19 using the suggested ensemble stacking technique, with percentages approaching 99%.
[43]	Chemo/ Bio-informatics	Special formulation for meningitis	The utilization of Ceftriaxone gelatin nanospheres or tripalmitin solid lipid nanoparticles has been proven to be a more practicable and effective nasal-to-brain formulation for the purpose of targeting meningitis in comparison to cefotaxime.
[19]	Chemoinformatics	Phytochemistry therapeutic discovery	The cytotoxic activity against HEPG2 and HUH-7 liver cancer cell lines attributed to the extract of *Eucalyptus globulus* bark was considerably high, and its absorption was found to be enhanced through the application of nanoformulation.
[46]	Chemoinformatics	Targeting Cancer Cells	Findings of the study demonstrate that ECT2 is capable of elevating both mRNA and protein concentrations in different types of human tumors, thereby enabling greater elimination of myeloid-derived suppressor cells (MDSC) and reducing the population of natural killer T (NKT) cells, resulting in a poor prognosis for survival. The investigation looked for medicines that could both inhibit ECT2 and function as anticancer agents.
[50]	Chemo/ Bio-informatics	Special formulation for Alzheimer disease	Curcumin outperformed bisdemethoxycurcumin (BDMC) in a nose-to-brain formulation for treatment of Alzheimer’s disease.
[18]	Chemo/ Bio-informatics	Testing Antibacterial activity against Resistant microorganisms	Three pyrazole and benzimidazole-based compounds examined showed modest bactericidal efficacy against MSSA, MRSA, and vancomycin-resistant Staphylococcus aureus (VRSA).
[34]	Bioinformatics	Genomic correlation with disease state	It was discovered that 12 SNPs were shared by the majority of the participants related to obesity and were concordant with their clinical diagnostics. In addition, results showed the presence of the mtDNA mutation A4282G in all samples; moreover, it is linked to chronic progressive external ophthalmoplegia
[39]	Bioinformatics	Multidrug-resistant organism identification	The highest represented genes among the 32 antimicrobial resistance genes discovered in pediatric cancer patients that exceeded the study threshold coverage were the aph(6)-Id gene, sul2, aph(3′)-Ia, sul1, dfrA12, aph(3″)-Ib, NDM-11, and TEM-220. Suggesting a horizontal transfer of resistance genes and plasmids between species in the context of nosocomial infections.
[51]	Cheminformatics	Hybrid Harris Hawks Optimization with Cuckoo Search	The experimental and statistical analyses demonstrate that the Hybrid Harris Hawks Optimization with Cuckoo Search method outperforms competitor algorithms.
[56,57,58,59,60,61]	Chemo/Bioinformatics	Integration with Artificial Intelligence	Different applications in molecular evolution, protein structure analysis, genomics for disease research, and system biology

## Data Availability

Not applicable.
